# Supplementary material for: The Systems Biology Research Tool: evolvable open-source software
Source: BMC Syst Biol. 2008 Jun 29;2:55. doi: 10.1186/1752-0509-2-55 (PMC2446383; doi:10.1186/1752-0509-2-55)
Supplement: Additional file 1 — SBRT Archive. An archive of the current version of the Systems Biology Research Tool. [file 1752-0509-2-55-S1.zip › sbrt-1.4.0/doc/developers_guide/api/sbrt/shell/proc/fba/PalssonRxnNodeParser.html]

PalssonRxnNodeParser


---


|  |  |  |
| --- | --- | --- |
| **Overview** \| **Package** \| **Class** \| **Use** \| **Tree** \| **Deprecated** \| **Index** \| **Help** | |  |
| **PREV CLASS**   **NEXT CLASS** | **FRAMES**    **NO FRAMES**     **All Classes** |  |
| SUMMARY: NESTED \| FIELD \| CONSTR \| METHOD | DETAIL: FIELD \| CONSTR \| METHOD |  |


---


## sbrt.shell.proc.fba Class PalssonRxnNodeParser

```
java.lang.Object
  sbrt.shell.proc.fba.PalssonRxnNodeParser
```

---

``` public class PalssonRxnNodeParser extends java.lang.Object ```

This class is used to make a direct translation from SBML 'reaction' nodes into
Systems Biology Research Tool objects. The names of reactions or chemical species
are not checked or processed in any way during this translation. This class works
for SBML files downloaded directly from Palsson's website.

**Author:**
:   This class was written and documented by Jeremiah Wright while in the
    Wagner lab.

---

| **Field Summary** | |
| --- | --- |
| `static java.lang.String` | `RXN_NODE_NAME`             The name of the nodes parsed by this class ('reaction'). |


**Constructor Summary**

`PalssonRxnNodeParser()`
  
          Constructs a new SBML reaction node parser.


| **Method Summary** | |
| --- | --- |
| `Or<And<java.lang.String>>` | `getCatalysts(org.w3c.dom.Node reactionNode, java.lang.String prefix)`             Returns the boolean logic rules for the catalysts of the provided reaction node. |
| `Interval` | `getFluxBounds(org.w3c.dom.Node reactionNode)`             Returns the flux bounds of the provided reaction node. |
| `double` | `getFluxValue(org.w3c.dom.Node reactionNode)`             Returns the flux value of the provided reaction node. |
| `Or<And<java.lang.String>>` | `getGenes(org.w3c.dom.Node reactionNode)`             Calls getCatalysts(Node, String) with "`GENE_ASSOCIATION:`" as the `prefix`. |
| `double` | `getLowerBound(org.w3c.dom.Node reactionNode)`             Returns the lower flux bound of the provided reaction node. |
| `LinearComb<java.lang.String>` | `getProducts(org.w3c.dom.Node reactionNode)`             Returns the products of the provided reaction node. |
| `Or<And<java.lang.String>>` | `getProteins(org.w3c.dom.Node reactionNode)`             Calls getCatalysts(Node, String) with "`PROTEIN_ASSOCIATION:`" as the `prefix`. |
| `LinearComb<java.lang.String>` | `getReactants(org.w3c.dom.Node reactionNode)`             Returns the reactants of the provided reaction node. |
| `java.lang.String` | `getRxnId(org.w3c.dom.Node reactionNode)`             Returns the reaction ID of the provided reaction node. |
| `double` | `getUpperBound(org.w3c.dom.Node reactionNode)`             Returns the upper flux bound of the provided reaction node. |
| `boolean` | `isReversible(org.w3c.dom.Node reactionNode)`             Indicates if the reaction in the provided reaction node is reversible. |

**Methods inherited from class java.lang.Object**

`clone, equals, finalize, getClass, hashCode, notify, notifyAll, toString, wait, wait, wait`

**Field Detail**

### RXN\_NODE\_NAME

```
public static final java.lang.String RXN_NODE_NAME
```

:   The name of the nodes parsed by this class ('reaction').

    **See Also:**: Constant Field Values


**Constructor Detail**

### PalssonRxnNodeParser

```
public PalssonRxnNodeParser()
```

:   Constructs a new SBML reaction node parser.


**Method Detail**

### getRxnId

```
public java.lang.String getRxnId(org.w3c.dom.Node reactionNode)
```

:   Returns the reaction ID of the provided reaction node.

    :   **Parameters:**: `reactionNode` - the reaction node whose ID is to be returned. **Returns:**: the reaction ID of the provided reaction node.

---


### getFluxBounds

```
public Interval getFluxBounds(org.w3c.dom.Node reactionNode)
```

:   Returns the flux bounds of the provided reaction node.

    :   **Parameters:**: `reactionNode` - the reaction node whose flux bounds are to be returned. **Returns:**: the flux bounds of the provided reaction node.

---


### getLowerBound

```
public double getLowerBound(org.w3c.dom.Node reactionNode)
```

:   Returns the lower flux bound of the provided reaction node.

    :   **Parameters:**: `reactionNode` - the reaction node whose lower flux bound is to be returned. **Returns:**: the lower flux bound of the provided reaction node.

---


### getUpperBound

```
public double getUpperBound(org.w3c.dom.Node reactionNode)
```

:   Returns the upper flux bound of the provided reaction node.

    :   **Parameters:**: `reactionNode` - the reaction node whose upper flux bound is to be returned. **Returns:**: the upper flux bound of the provided reaction node.

---


### getFluxValue

```
public double getFluxValue(org.w3c.dom.Node reactionNode)
```

:   Returns the flux value of the provided reaction node.

    :   **Parameters:**: `reactionNode` - the reaction node whose flux value are to be returned. **Returns:**: the flux value of the provided reaction node.

---


### getReactants

```
public LinearComb<java.lang.String> getReactants(org.w3c.dom.Node reactionNode)
```

:   Returns the reactants of the provided reaction node.

    :   **Parameters:**: `reactionNode` - the reaction node whose reactants are to be returned. **Returns:**: the reactants of the provided reaction node.

---


### getProducts

```
public LinearComb<java.lang.String> getProducts(org.w3c.dom.Node reactionNode)
```

:   Returns the products of the provided reaction node.

    :   **Parameters:**: `reactionNode` - the reaction node whose products are to be returned. **Returns:**: the products of the provided reaction node.

---


### isReversible

```
public boolean isReversible(org.w3c.dom.Node reactionNode)
```

:   Indicates if the reaction in the provided reaction node is reversible.

    :   **Parameters:**: `reactionNode` - the reaction node whose reversibility is to be determined. **Returns:**: `true` if the reaction node indicates a reversible reaction; `false` if the reaction node indicates an irreversible reaction.

---


### getGenes

```
public Or<And<java.lang.String>> getGenes(org.w3c.dom.Node reactionNode)
```

:   Calls getCatalysts(Node, String) with "`GENE_ASSOCIATION:`"
    as the `prefix`.

    :   **Parameters:**: `reactionNode` - the reaction node for which the genes are to be returned. **Returns:**: the boolean logic rules for the genes of the provided reaction node.

---


### getProteins

```
public Or<And<java.lang.String>> getProteins(org.w3c.dom.Node reactionNode)
```

:   Calls getCatalysts(Node, String) with "`PROTEIN_ASSOCIATION:`"
    as the `prefix`.

    :   **Parameters:**: `reactionNode` - the reaction node for which the proteins are to be returned. **Returns:**: the boolean logic rules for the proteins of the provided reaction node.

---


### getCatalysts

```
public Or<And<java.lang.String>> getCatalysts(org.w3c.dom.Node reactionNode,
                                              java.lang.String prefix)
```

:   Returns the boolean logic rules for the catalysts of the provided
    reaction node.

    :   **Parameters:**: `reactionNode` - the reaction node for which the catalysts are to be returned.: `prefix` - the string used to denote these catalysts in the SBML file. **Returns:**: the boolean logic rules for the catalysts of the provided reaction node.


---


|  |  |  |
| --- | --- | --- |
| **Overview** \| **Package** \| **Class** \| **Use** \| **Tree** \| **Deprecated** \| **Index** \| **Help** | |  |
| **PREV CLASS**   **NEXT CLASS** | **FRAMES**    **NO FRAMES**     **All Classes** |  |
| SUMMARY: NESTED \| FIELD \| CONSTR \| METHOD | DETAIL: FIELD \| CONSTR \| METHOD |  |


---
